# The Sunflower as a Preventive of Miasma

**Published:** 1875-12

**Authors:** 


The Sunflower as a Preventive of Miasma.—A correspondent of the *Soil of the South,* writing from a place in Alabama, which he says was peculiarly subject to fevers, gives the result of his experience in the premises, and in not a single instance where he planted sunflowers around his negro cabins did their inmates suffer from fevers; while his wife, two children, and two house servants, all had fevers, he not having planted any of the sunflowers around his own dwelling, which, in his opinion, accounts for the difference in the results.—*The Medical Record.*

